# Higher intrinsic network excitability in ventral compared with the dorsal hippocampus is controlled less effectively by GABA_B_ receptors

**DOI:** 10.1186/s12868-015-0213-z

**Published:** 2015-11-10

**Authors:** Costas Papatheodoropoulos

**Affiliations:** Laboratory of Physiology, Department of Medicine, School of Health Sciences, University of Patras, Rion, 26504 Patras, Greece

**Keywords:** Hippocampus, Septotemporal, Dorsoventral, Excitability, Inhibition, Disinhibition, Network, GABA_A_ receptor, GABA_B_ receptor, NMDA receptor

## Abstract

**Background:**

Elucidating specializations of the intrinsic neuronal network between the dorsal and the ventral hippocampus is a recently emerging area of research that is expected to help us understand the mechanisms underlying large scale functional diversification along the hippocampus. The aim of this study was to characterize spontaneous network activity between the dorsal and the ventral hippocampus induced under conditions of partial or complete blockade of GABAergic inhibition (i.e. disinhibition).

**Results:**

Using field recordings from the CA3 and CA1 fields of hippocampal slices from adult rats I found that ventral compared with dorsal hippocampus slices displayed higher propensity for and higher frequency of occurrence of spontaneous field potentials (spfps) at every level of disinhibition. Also NMDA receptor-depended spfps (spfps_-nmda_) occurred with higher probability more frequently and were larger in the ventral compared with the dorsal hippocampus. Importantly, blockade of GABA_B_ receptors produced a stronger effect in enhancing the probability of generation of spfps and spfps_-nmda_ in the dorsal compared with the ventral hippocampal slices and increased spfps_-nmda_ only in dorsal slices.

**Conclusion:**

These results demonstrate a higher intrinsic neuronal excitability of the ventral compared with the dorsal local circuitry with the considerable contribution of NMDA receptors. Furthermore, the GABA_B_ receptors control the total and the NMDA receptor-dependent excitation much less effectively in the ventral part of the hippocampus. It is proposed that NMDA and GABA_B_ receptors significantly contribute to differentiate local network dynamics between the dorsal and the ventral hippocampus with important implications in the information processing performed along the long hippocampal axis.

## Background

Local neuronal networks participate to brain functions by changing their excitability status through a dynamic modulation of the balance between excitation and inhibition [1]. The intrinsic hippocampal circuitry has been perceived as a model to understand the interactions between the various components that control the excitation/inhibition balance in local circuitry. Continuous experimental work for decades has accumulated a great amount of information about the function of intrinsic neuronal circuitry of the hippocampus [2]. The internal circuitry of the hippocampus presents some very notable regularity, in that its fundamental structural organization is consisted of a local “trisynaptic” circuitry repeated along the long (dorsoventral or septotemporal) axis of the structure as a regular unit [3]. The longitudinal repetition of this “canonical module” has created the persistent view of the hippocampus as a structure with homogeneity along its dorsoventral course. For long time the idea of this dorsoventral internal homogeneity of the hippocampal circuitry has neglected early neurochemical and physiological evidence showing differences along the hippocampus [4–10]. Only recently the differences along the dorsoventral axis and especially between the DH and VH have been more systematically attracted the attention of researchers. For instance, differences between DH and VH have been revealed in the synaptic transmission and neurotransmitter receptors [11–14] as well as in the synaptic plasticity [15–18]. Yet, the first characteristic difference that was observed between the two hippocampal poles was the higher susceptibility of the ventral hippocampus in rodents and the anterior hippocampus in human to epileptic activities [19, 20]. These early observations were repeatedly confirmed and extended by subsequent more thorough in vivo and in vitro studies performed in rats [4, 7, 21–30]. These studies led to the idea of the greater overall excitability of VH compared with DH. However, the mechanisms of apparent higher excitability of VH are still not well understood, perhaps because differences in network excitability arise from a combination of molecular, cellular and network mechanisms. GABAergic synaptic transmission constitutes a basic determinant of the local neuronal network excitability [31]. A typical experimental approach to study synchronous network activity is based on the induction of spontaneous activity in isolated brain preparations [32]. Classically, this is achieved by changing the excitation/inhibition balance through lowering inhibitory activity using the so-called disinhibition models [33]. Indeed, GABAergic transmission could be a crucial factor in determining the threshold for excitation of the local circuit [34]. Paradoxically, however, the effects of blockade of GABAergic transmission on the spontaneous network activity have been never examined comparatively between DH and VH. In the present study, I comparatively examined the ability of DH and VH to spontaneously generate population activity under conditions of partial or complete disinhibition using field recordings from transverse hippocampal slices. The results show that the intrinsic neuronal network of VH compared with DH exhibits higher excitability with the NMDA receptors having a significant contribution in the higher excitability of VH. Furthermore, GABA_B_Rs play a more important role in restricting network excitability in DH than in VH.

## Methods

### Animals and slice preparation

**Fig. 1:**
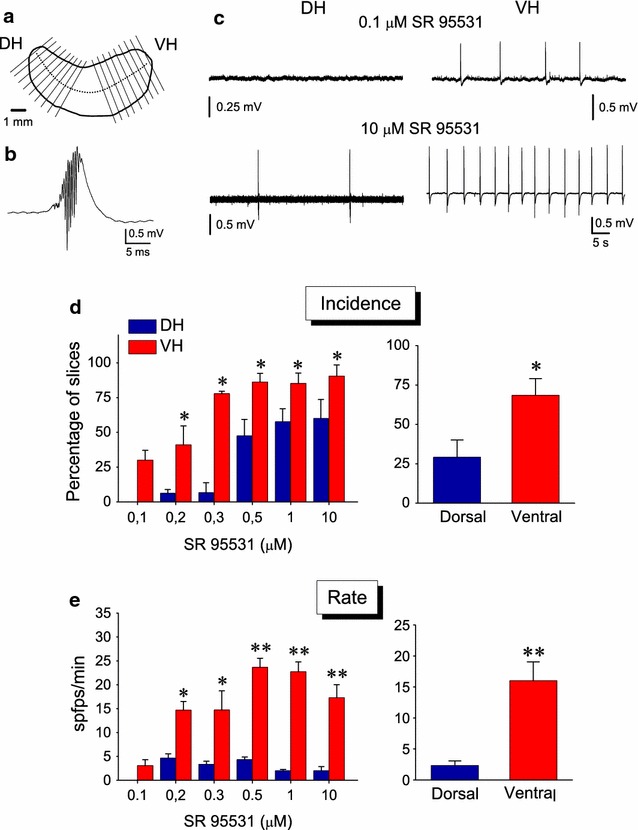
Higher incidence and rate of disinhibition-induced spfps in VH compared with DH. **a** Schematic drawing of the hippocampus where the cutting plane directions and the extent of sliced structure are indicated by the *solid lines*. *Dotted line* indicates the longitudinal axis of the structure. **b** Fast sweep speed record of a single spontaneous field potential (spfp) induced under blockade of GABA_A_Rs by their antagonist SR 95531. Recording was made in the CA3 stratum pyramidale of a VH slice. **c** Continuous recordings from the CA3 stratum pyramidale illustrating that at the low concentration of 0.1 μM the antagonist of GABA_A_Rs SR 95531 induced spfps in VH but not DH. Even at the highest concentration of SR 95531 used (10 μM) the rate of spfps was higher in VH than in DH. **d** Histogram of percentages of DH and VH slices displaying spfps under different concentrations of SR 95531 (plot on the *left*) and the cumulative results for all concentrations (plot on the *right*) are shown. The number of slices examined under the six different drug concentrations are given in the main text. **e** Histogram of the rate of spfps in DH and VH under the various SR 95531 concentrations and the cumulative mean values for all concentrations are shown in the *left* and *right* graph, respectively. *Asterisks* in all graphs denote statistically significant differences between DH and VH at *p < 0.05 or **p < 0.01 (Mann–Whitney *U* test). Statistical comparison at 0.1 μM was not possible because of the absence of spfps in DH slices

Forty-five adult (2–4 months old) male Wistar rats (Athens Pasteur Institute) were used in this study. Animals were obtained from the Animal Facility of the Medical School of the University of Patras. All experimental treatment and procedures were conducted in accordance with the European Communities Council Directive Guidelines (86/609/EEC, JL 358, 1, December, 12, 1987) for the care and use of Laboratory animals and they have been approved by the Prefectural Animal Care and Use Committee (No: EL 13BIO04). In addition, all efforts have been made to minimize the number and the suffering of animals used. Animals were housed in a room with a controlled light–dark cycle (12 h light–12 h dark) and free access to food and water. Slices from the two hippocampal poles were prepared as follows. The animals after deep anesthesia with diethyl ether were decapitated with a guillotine and their brain was removed and submerged in chilled (2–4 °C) standard medium containing (in mM): 124 NaCl; 4 KCl; 2 MgSO_4_; 2 CaCl_2_; 1.25 NaH_2_PO_4_; 26 NaHCO_3_; 10 glucose; at pH 7.4, and the two hippocampi were excised free. Using a McIlwain tissue chopper, 500–550 µm thick slices, transverse to the long axis of hippocampus, were prepared from the ventral (temporal) and the dorsal (septal) parts of the structure. In particular, slices were prepared from the regions extending between about 0.1 and 4.5 mm from the dorsal and the ventral ends of the hippocampus (Fig. 1a). These slices will be referred to as dorsal (DH) and ventral (VH) hippocampal slices. In each experiment, slices from both DH and VH from the same hippocampus or from the hippocampi of the same animal were used. In particular, 227 DH and 233 VH slices were studied. Immediately after sectioning, the slices were transferred to an interface type recording chamber where they were maintained at a constant temperature of 31 ± 0.5 °C, continuously humidified with a mixed gas containing 95 % O_2_ and 5 % CO_2_ and perfused with standard artificial cerebrospinal fluid.

### Recordings, data processing and analysis

Extracellular field recordings from the CA3 or CA1 fields were made using carbon fiber electrodes (diameter 5–10 µm, Kation Scientific, or World Precision Instruments Inc., USA). Recordings started at about 1.5 h after the slices were placed in the recording chamber and they were made either from the CA3 stratum pyramidale or from the CA1 stratum radiatum, as explained in the Results’ section. Signals were acquired with a Neurolog amplifier (Digitimer Limited, UK), band-pass filtered at 0.5 Hz–2 kHz, digitized at 10 kHz and stored in a computer disk using the CED 1401-plus interface and the Signal6 or the Spike2 software (Cambridge Electronic Design, Cambridge, UK) for off-line analysis. Spontaneous field potentials (spfps) and NMDAR-mediated spontaneous field potentials (spfps_-nmda_) were quantified by their incidence, measured as the percentage of slices displaying spontaneous activity in the population of slices studied; the rate of their occurrence, measured as the number of events per minute. Spfps_-nmda_ were also quantified by their area, measured as the area circumscribed by the waveform of the negative field potential and the baseline.

### Drugs

The following drugs were used: the antagonist of ionotropic non-NMDARs 6-Cyano-7-nitroquinoxaline-2,3-dione (CNQX), the antagonist of NMDARs 3-((*R*)-2-carboxypiperazin-4-yl)-propyl-1-phosphonic acid (CPP), the non-competitive antagonist of NMDARs (5*S*,10*R*)-(+)-5-methyl-10,11-dihydro-5*H*-dibenzo[a,d]cyclohepten-5,10-imine maleate (MK 801), the antagonists of γ-amino-butyric acid type A receptors (GABA_A_R) SR 95531, the GABA_A_R’s channel blocker picrotoxin (PTX), the antagonist of GABA_B_Rs (3-aminopropyl)(diethoxymethyl) phosphinic acid (CGP 35348), and the agonist of adenosine A_1_ receptors 2-chloroadenosine (adenosine). All drugs were purchased from Tocris Cookson (UK), but PTX and adenosine were obtained from Sigma (USA). They were first dissolved in water, but CNQX was dissolved in dimethyl-sulfoxide (DMSO). The v/v concentration of DMSO at the final solution was lower than 0.05 %.

### Statistics

The non parametric Wilcoxon and Mann–Whitney *U* test were used for comparisons inside and between DH and VH groups of values, respectively. For comparison of percentages the χ^2^ test was also used. ANOVA was used for comparison between related multiple groups of data. Values throughout the text represent mean ± SEM; “n” and numbers into parenthesis indicate the number of slices studied.

## Results

### Local network excitation is higher and less effectively controlled by GABA_B_Rs in VH compared with DH

**Fig. 2:**
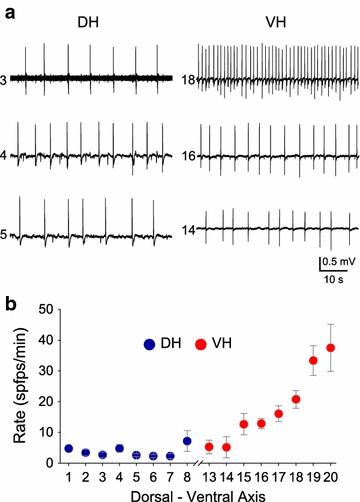
The rate of spfps correlates with the position of the slice along the ventral but not the dorsal hippocampus. **a** Example recordings illustrating that slices taken from more extreme locations in the VH but not the DH displayed higher rates of spfps. The number on the *left* of each trace indicates the position of the slice along the dorsoventral axis of the hippocampus. **b** Cumulative graph showing that the rate of spfps was linearly correlated with the distance of the slice from the ventral but not the dorsal end of the hippocampus. The *numbers* in abscissa correspond to the successive locations along the dorsoventral axis of the structure that slices were taken from. Thus, about twenty positions for 500–550 μm thick slices can be allocated along the entire rat hippocampus which is 10–11 mm long. The medial part of the hippocampus (i.e. locations 9–12) is not shown for clarity reasons. Data were obtained from all SR 95531 concentrations. Only slices that displayed spfps were included in the analysis. The ANOVA test showed that the rate of spfps differed along the VH (F = 6.29, p < 0.001) but not along the DH

Spontaneous field potentials (spfps) induced under conditions of blockade of GABA_A_Rs (Fig. 1b) were comparatively studied in DH and VH slices (Fig. 1c). GABA_A_R-mediated transmission was reduced by applying different concentrations of the antagonist of GABA_A_R SR 95531, from 0.1 to 10 μM. The incidence of spfps in the slice population (i.e. the percentage of slices displaying spfps) and the rate of spfps in each drug concentration were measured in DH and VH. Perfusing slices with increasing concentrations of SR 95531 induced the appearance of spfps in a, respectively, increasing number of slices, in both DH and VH. Furthermore, the incidence of spfps was very different between DH and VH at all concentrations of SR 95531 used (Fig. 1d). In particular, the incidence of spfps at the drug concentrations of 0.1, 0.2, 0.3, 0.5, 1 and 10 μM were 0 % (15), 6.3 ± 2.8 % (32), 11.8 ± 7.1 % (17), 47.5 ± 11.8 % (66), 57.6 ± 9.4 % (57) and 60.0 ± 13.6 % (35), respectively, in DH and 30.0 ± 7.1 % (20), 41.0 ± 13.7 % (31), 77.8 ± 1.8 % (18), 86.2 ± 6.2 % (56), 85.2 ± 7.5 (37) and 90.4 ± 8.0 % (32), respectively, in VH. The difference in incidence between DH and VH was significantly different at every drug concentration (Mann–Whitney *U* test and χ^2^ test, p < 0.05). The calculation of the total incidence’s values (i.e. taking into account all drug concentrations) gave 29.1 ± 11.0 % for DH and 68.4 ± 10.6 % for VH (Mann–Whitney *U* test and χ^2^ test, p < 0.05). The rate of spfps was several folds higher in VH compared with DH at all drug concentrations (Fig. 1e). In particular, the rate of spfps at the drug concentrations of 0.1, 0.2, 0.3, 0.5, 1 and 10 μM were 0, 4.7 ± 0.9 % (2), 3.4 ± 0.6 % (2), 4.3 ± 0.6 % (25), 2.0 ± 0.3 % (30) and 2.0 ± 0.9 % (20), respectively, in DH and 3.1 ± 1.3 % (6), 14.7 ± 1.8 % (12), 14.8 ± 4.1 % (13), 23.7 ± 1.9 % (48), 22.7 ± 2.1 (26) and 17.3 ± 2.7 % (29), respectively, in VH. The difference in the rate of spfps between DH and VH was significantly different at every drug concentration (Mann–Whitney *U*-test, p < 0.05 or p < 0.01, see Fig. 1e). The overall rate of spfps (i.e. taking into account all drug concentrations) was 2.3 ± 0.7 spfps/min for DH and 16.0 ± 3.0 spfps/min for VH (Mann–Whitney *U* test, p < 0.01). An interesting observation was that the rate of spfps was significantly correlated with the precise location of the VH but not DH slices in the long axis of the structure. As shown in Fig. 2, the rate of spfps was significantly higher in slices taken from more extreme compared with more medial positions in the VH but not the DH, regardless the concentration of SR 95531. Specifically, the rate of spfps increased from about 5 spfps/min in slices located 3.5–4.0 mm from the ventral end to about 35 spfps/min in the slices obtained from the extreme 1.0 mm of the VH (ANOVA, F = 6.29, p < 0.001). On the contrary, the rate of spfps was quite comparable among slices obtained from the DH (ANOVA, F = 1.85, p > 0.1).

**Fig. 3:**
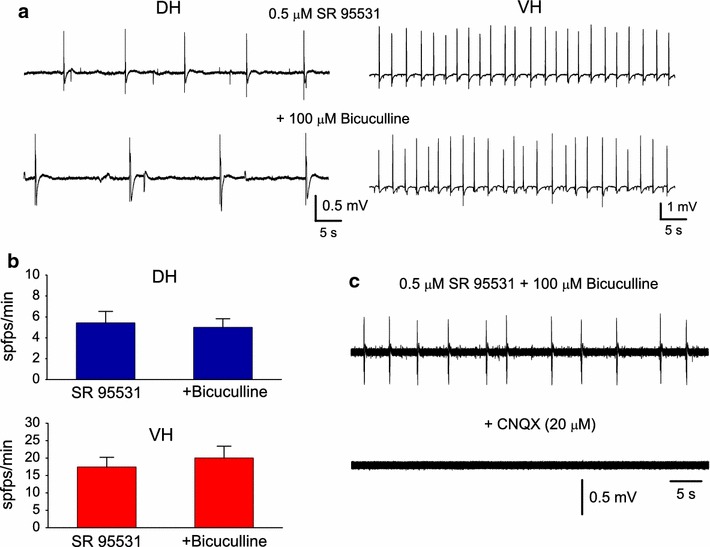
Addition of bicuculline to slices with ongoing spfps does not affect the generation of spfps. **a** Continuous 1 min recordings from a dorsal and a ventral slice during the application of 0.5 μM SR 95531 and during the additional application of 100 μM bicucullline are shown. **b** Cumulative results on the rate of spfps obtained from seven dorsal and nine ventral slices. **c** Example recording illustrating that spfps induced by SR 95531 were abolished by the antagonist of non-NMDARs CNQX

In a set of experiments the effects of high concentration of bicuculline (100 μM) was examined in slices perfused with SR 95531 and generating spfps. Addition of bicuculline did not significantly change the rate of spfps either in DH or VH slices. Specifically, the rate of spfps under SR 95531 and following addition of bicuculline was 5.4 ± 1.1 spfps/min and 5.0 ± 0.8 spfps/min, respectively, in DH (a change of 1.69 ± 17.0 %, n = 7, Wilcoxon test, p > 0.05), and 17.5 ± 2.8 spfps/min and 20.0 ± 3.4 spfps/min, respectively, in VH (a change of 12.6 ± 8.5 %, n = 10, Wilcoxon test, p > 0.05), (Fig. 3a, b). Spfps were abolished upon addition of 20 μM CNQX (n = 3, Fig. 3c) indicating that they required the activity of non-NMDARs.

**Fig. 4:**
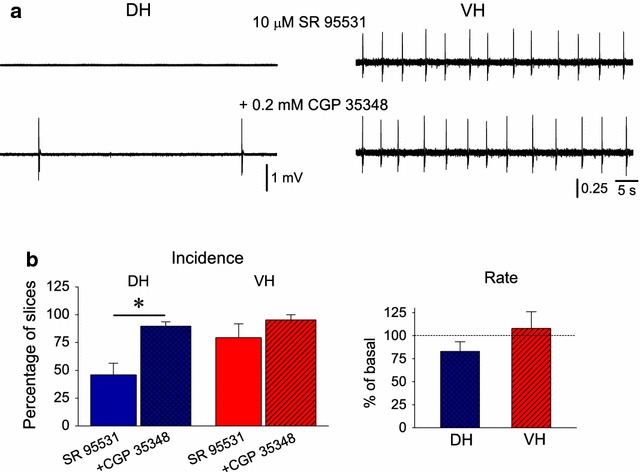
GABA_B_Rs control the likelihood of spfps generation in DH but not VH. **a** Example recordings illustrating that blockade of GABA_B_Rs by CGP 35348 (200 μM) induced spfps in a previously silent dorsal slice without affecting the rate of ongoing spfps in another ventral slice. **b** Cumulative results on the incidence and the rate of spfps are shown. *Asterisk* indicate statistically significant drug effect at p < 0.05 (Wilcoxon test)

In addition to GABA_A_Rs, the GABA_B_R-mediated transmission plays an important role in controlling neuronal activity and network excitability [35]. Thus, I examined whether blockade of GABA_B_Rs could further enhance excitability in DH and VH by applying CGP 35348 (200 μM) in slices already perfused with 10 μM SR 95531. I found that blockade of GABA_B_Rs induced a considerable increase in the incidence of spfps in DH (from 45.9 ± 10.5 to 89.6 ± 4.0 %, n = 24, Wilcoxon test and χ^2^ test, p < 0.05) but not in VH (from 79.4 ± 12.4 to 95.3 ± 4.8 %, n = 9, Wilcoxon test and χ^2^ test, p > 0.05), (Fig. 4). Regarding VH, the blockade of GABA_B_Rs could not have considerable effect since the incidence of spfps in VH slices was already very high under SR 95531. CGP 35348 did not significantly change the rate of spfps either in DH (from 0.7 ± 0.1 to 0.6 ± 0.1 spfsp/min, n = 11) or VH (from 15.0 ± 4.3 to 16.2 ± 5.7 spfsp/min, n = 7), (Fig. 4b). In order to examine whether there is any difference in spfps between DH and VH under blockade of only GABA_B_Rs, a population of ten dorsal and eight ventral slices taken from three animals were bathed with CGP 35348 alone. However, the drug did not induce any detectable synchronous field activity in any of the slices studied.

### NMDAR-depended network excitation is higher and weakly controlled by GABA_B_Rs in VH compared with DH

**Fig. 5:**
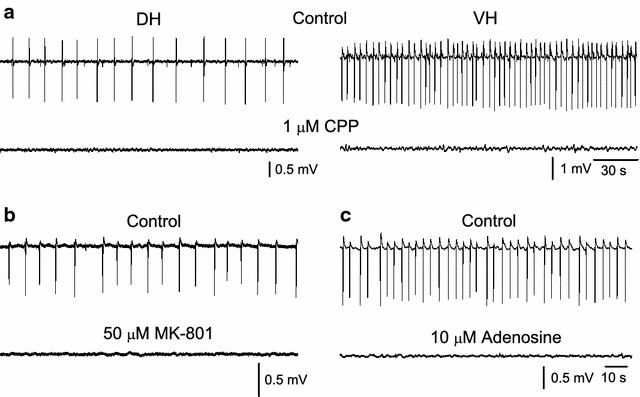
Dependence of spfps_-nmda_ on NMDARs and glutamate release. Example recordings illustrating that spfps_-nmda_ were completely abolished by the competitive antagonist of NMDARs CPP (**a**) or the open channel blocker of the same receptors MK-801 (**b**). Spfps_-nmda_ were also blocked by activation of adenosine A_1_ receptors by their agonist 2-chloroadenosine (**c**). Data shown in “**b**” and “**c**” were obtained from two DH slices

**Fig. 6:**
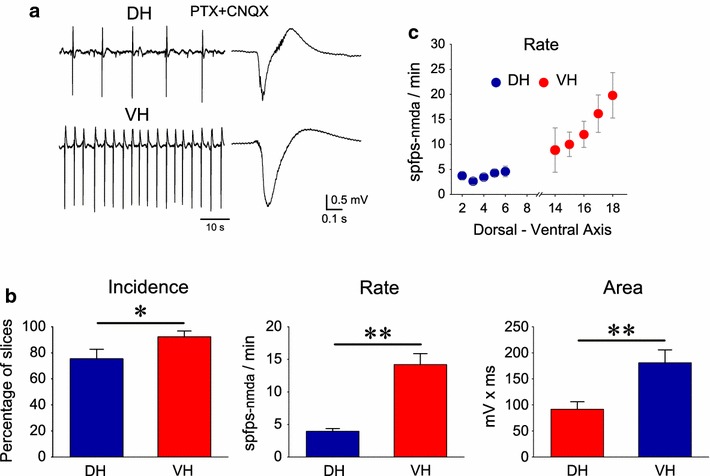
Higher incidence, rate and area of spfps_-nmda_ in VH compared with DH. **a** Simultaneous recordings of spfps_-nmda_ from the CA1 stratum radiatum of a DH and a VH slice taken from the same animal and bathed in 50 μM PTX and 20 μM CNQX. One minute-long records (traces on the *left*) and single events (traces on the *right*) are shown. Note that spfps_-nmda_ was larger and occurred at a higher rate in VH compared with the DH slice. **b** The incidence, rate and area of spfps_-nmda_ were significantly greater in VH compared with DH slices. *Asterisks* denote statistical significance between DH and VH values (Mann–Whitney *U* test test, at *p < 0.05 or **p < 0.005). **c** Diagram illustrating the relationship between the rate of occurrence of spfps_-nmda_ and the slice position along the dorsoventral axis of the hippocampus. Only slices taken from the VH displayed positive correlation in their rates of spfps_-nmda_ as approached the end of the hippocampus structure (F = 2.5, p < 0.05). Note that slices taken from the most extreme positions did not show spfps_-nmda_. The medial part of the hippocampus (i.e. locations 9-13) is not shown for clarity

NMDARs are a very important component of the excitatory glutamatergic transmission in the hippocampus [36]. Among pyramidal cells those located in the CA1 field display especially large amounts of NMDARs [37]. Recently, it has been shown that NMDARs differ between DH and VH in their subunit composition [11], involvement in high-frequency oscillations [38], epileptogenesis [27, 28] and participation to short-term synaptic plasticity [39]. In order to examine the relative participation of NMDARs in the network excitability comparatively between DH and VH, slices were studied under conditions that promote the activity of NMDARs. In particular, slices were bathed with free-magnesium medium containing the blocker of ion channel of GABA_A_Rs PTX (50 μM) and the antagonist of non-NMDARs CNQX (20 μM). Taking into account that NMDARs are predominately located in dendrites [40] recordings were made from the CA1 stratum radiatum. Under these conditions spfps were induced in both DH and VH slices. These potentials were completely abolished upon perfusion with the antagonist of NMDARs CPP (1–10 μM) in both DH (n = 13) and VH slices (n = 16) or the NMDAR’s open channel blocker MK-801 (50 μM, in 5 DH and 5 VH slices), (Fig. 5a, b); therefore they will be called spfps_-nmda_. Spfps_-nmda_ were also suppressed following activation of adenosine A_1_ receptors by 6-chloroadenosine (5–10 μM), (Fig. 5c). Given that A_1_ receptors in CA1 control the neurotransmitter release from glutamatergic terminals [41, 42] these observations showed that the generation of spfps_-nmda_ required the activation of postsynaptic NMDARs from glutamate released from presynaptic terminals. Most of the DH and VH slices displayed spfps_-nmda_ (Fig. 6a). Furthermore, their incidence was significantly greater in VH (92.3 ± 4.5 % of slices, 61 slices studied) than in DH (75.4 ± 7.5 % of slices, 69 slices studied, comparison between DH and VH; Mann–Whitney *U* test and χ^2^ test, p < 0.05), (Fig. 6b). In addition, and in similarity with spfps, spfps_-nmda_ occurred at a much faster rate in VH (13.2 ± 1.5 events/min, n = 55) compared with DH (3.7 ± 0.4 events/min, n = 54), (Mann–Whitney *U* test, p < 0.001, Fig. 6c). Furthermore, spfp-_nmda_ were significantly greater in VH than in DH. Specifically, the area of single spfps_-nmda_ was two-fold higher in VH (180.8 ± 25.0 mV × ms, n = 44) compared with DH slices (91.8 ± 14.3 mV × ms, n = 32), (Mann–Whitney *U* test, p < 0.001). This is consistent with the higher extracellular concentration of glutamate in VH compared with DH [8, 43], given that ambient glutamate activates NMDARs in CA1 pyramidal cells [44].

In similarity with spfps, the rate of spfps_-nmda_ was a function of the precise position of slice in VH but not DH (Fig. 6c). Specifically, VH slices taken from progressively more extreme positions, toward the ventral end of the structure, displayed higher rates of spfps-nmda than slices obtained from more medial positions (ANOVA, F = 2.5, p < 0.05), (Fig. 6b). Such a correlation was not observed in DH slices (ANOVA, F = 0.87, p > 0.5).

**Fig. 7:**
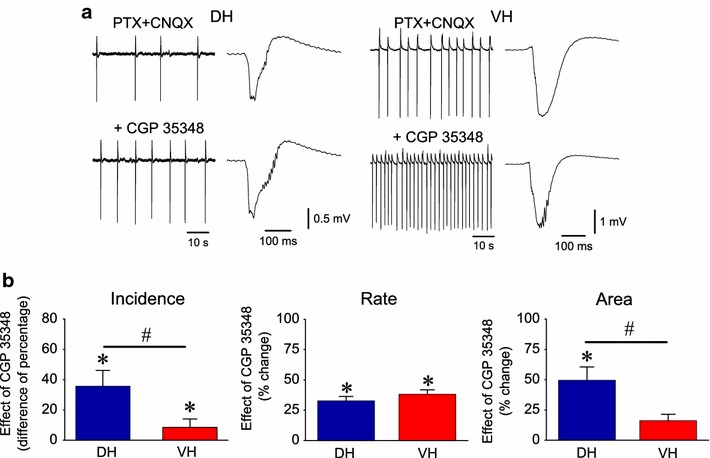
GABA_B_Rs control the incidence and the area of spfps_-nmda_ more in DH than in VH. **a** Examples of CA1 stratum radiatum recordings from dorsal and ventral slices before and during application of the antagonist of GABA_B_Rs CGP 35348 (200 μM). One-minute long records (traces on the *left*) and single events (traces on the *right*) from each slice are shown. Note that although the rate of spfps_-nmda_ was similarly accelerated by CGP 35348 in the dorsal and ventral slices, single events became larger only in the dorsal slice. **b** Cumulative data showing that blockade of GABA_B_Rs increased the incidence and area of spfps_-nmda_ more in DH than in VH. The effect of CGP 35348 on the incidence is given as the difference in the percentage of slices displaying spfps_-nmda_ before and after application of CGP 35348. *Asterisks* indicate statistically significant drug effects at p < 0.05 in each group (Wilcoxon test). *Diesis* indicates significant differences in the drug effect between DH and VH (Mann–Whitney *U* test, p < 0.05)

GABA_B_R-mediated transmission exerts a powerful control on NMDARs [45, 46]. Thus I examined the involvement of GABA_B_ receptors in the generation of spfp-_nmda_. The GABA_B_R antagonist CGP 35348 was applied to DH and VH slices perfused with PTX and CNQX (Fig. 7a). Blockade of GABA_B_Rs increased the incidence of spfp-_nmda_ in the population of both DH and VH slices. In particular, of the previously silent slices sixteen out of seventeen dorsal (94.5 ± 5.6 %) and all five ventral slices (100 ± 0 %) exhibited spfp-_nmda_ following addition of CGP 35348. The difference in the percentage of slices displaying spfps_-nmda_ before (i.e. under PTX + CNQX) and following addition of CGP 35348 was significantly higher in DH (35.8 ± 10.4 %) than in VH (8.5 ± 5.6 %; Mann–Whitney *U* test and χ^2^ test, p < 0.05; Fig. 7b). As a consequence, the incidence of spfps_-nmda_ observed under all three drugs (i.e. PTX + CNQX + CGP 35348) became similar between DH (68 out of 69 slices, 98.6 %) and VH (61 out of 61 slices, 100 %). Blockade of GABA_B_Rs significantly increased in the rate of spfp-_nmda_ similarly in DH (by 32.7 ± 3.7 %, from 4.2 ± 0.5 to 6.1 ± 0.6 events/min, n = 38, p < 0.001) and VH (by 38.2 ± 3.5 %, from 13.3 ± 1.8 to 17.6 ± 1.8 events/min, n = 39, p < 0.001), (Fig. 7b). Importantly, CGP 35348 produced a significant increase in the area of single spfp-_nmda_ in DH (by 49.5 ± 11 %, from 70.8 ± 14.5 to 102.2 ± 22.8 mV × ms, n = 20, Wilcoxon test, p < 0.05) but not VH (by 16.1 ± 5.3 %, from 186.8 ± 28.1 to 192.3 ± 24.2 mV × ms, n = 33, p > 0.05), (Fig. 7b). However, the area of single spfp-_nmda_ remained significantly greater in VH compared with DH slices (192.3 ± 24.2 vs 102.2 ± 22.8 mV × ms, Mann–Whitney *U* test, p < 0.01).

## Discussion

This study shows that under blockade of GABAergic transmission the local neuronal circuitry of the hippocampus is more excitable in the ventral than its dorsal segment with the considerable participation of NMDARs. Furthermore, the relatively weaker control of excitation by GABA_B_Rs appears to play an important role in the higher proneness of the ventral hippocampus to spontaneous activation.

One well proven difference between the DH and VH neuronal network is the propensity of the later to epileptic activity [4, 7, 19–30]. A characteristic expression of the particular excitability of VH compared with the DH is the higher rate of occurrence of recurrent synchronous discharges, observed especially in the in vitro hippocampal preparations under conditions of increased potassium or decreased magnesium concentration in the extracellular medium [4, 22, 26–28]. In keeping with these observations and using for the first time the experimental model of gradually increased disinhibition it is shown that the VH compared with DH displays higher rates of spfps and spfp_-nmda_. In addition, in the present study the higher network excitability of VH was also expressed by the greater incidence of spfps and spfps_-nmda_ and the larger area of spfps_-nmda_ under intact GABA_B_R-mediated inhibition. In particular, the present results show that the intrinsic circuitry of VH is more prone to spontaneous activation at any level of gradual reduction of GABA_A_R-mediated transmission.

Several mechanisms have been proposed to contribute to the higher intrinsic excitability in the VH, including the excitatory and the inhibitory synaptic actions. For instance, in the CA1 field of the VH vs DH the GABA_A_R-mediated transmission is lower as evidenced by the impact of inhibitory recurrent circuit on the firing of principal cells [12, 13] and the different composition of GABA_A_Rs [14]. Furthermore, the different subunit composition of NMDARs [11] and their different participation in the epileptogenesis between the two hippocampal poles [27, 28] has suggested that NMDARs are functionally distinct between DH and VH. Cholinergic transmission may also play a significant role in the network excitability [26]. Apparently, a plethora of molecular, synaptic, cellular and circuit mechanisms contribute to the different excitability of the local neuronal circuitry between DH and VH. One of the major factors contributing to increased network excitability is the relatively low effectiveness of the synaptic inhibition [47]. The disinhibition model used in the present study presents some advantages, including the fact that neuronal excitation that lead to synchronized field potentials can be studied in isolation from the confounding mechanisms of synaptic inhibition [33]. Hence, the higher rates of spontaneous network activation observed here under blockade of GABAergic inhibition suggest that the relative contribution of the neuronal excitation in the local neuronal circuitry is higher in the VH compared with the DH. Indeed, recent observations have shown that the pyramidal neurons of the ventral hippocampus are more excitable compared with their dorsal counterparts [48, 49].

GABA_B_Rs modulate network excitation [35] and especially NMDAR-depended activity [45, 46]. The fact that blockade of GABA_B_Rs increased the proportion of slices that displayed either spfps or spfps_-nmda_, suggests that GABA_B_R-mediated transmission regulates the excitation threshold in the local circuitry as for example occurs in the subiculum [34] and control synchronous discharges [50]. Importantly, the present results show that GABA_B_Rs effectively control the overall network excitability in DH but not VH. What is more, thought GABA_B_Rs restricted NMDAR-depended network excitation in both DH and VH, this effect was considerably stronger in DH. This is the first time that GABA_B_Rs are shown to be differently involved in regulating and controlling total and NMDAR-depended network excitability between DH and VH.

The intrinsic excitability of a local neuronal network is the expression of the balance between excitation and inhibition that dynamically tunes the function and therefore the output of a local neuronal circuit into a specific mode at any given instance. It is proposed that NMDARs and GABA_B_Rs play significant roles in sculpting this balance in DH and VH. It has been shown that partial blockade of inhibition leads to network oscillations [33]. It is noted that spontaneous activation of the VH local network occurred even at small reductions in GABA_A_Rergic inhibition induced by small concentrations of SR 95531. Small reductions of GABAergic inhibition such as those used in the present study favor the activity of sharp wave—ripples [51] which is the only one physiological endogenous network activity of the hippocampus. Actually, the generation of the ripple oscillation requires an accurate balance between excitation and inhibition in the local circuitry [52]. The higher excitability of the VH implies that physiological activation of the hippocampal network may arise from this part of the hippocampus. This should have important implications for the processing of the incoming information in the hippocampal circuitry. Indeed, the activity of sharp waves is self-organized with greater probability in ventral compared with dorsal hippocampal slices [53]. Similarly, sharp waves first emerge in the ventral segment of the hippocampus and then spread toward its dorsal end or they remain localized in the ventral pole [54]. It is then postulated that the higher network excitability of VH is the manifestation of the particular functional demands assigned to this part of the hippocampus.

## Conclusion

The present results show that GABA_B_Rs and NMDARs importantly contribute to the higher intrinsic excitability of the VH compared with the DH local circuitry. The higher excitability of the VH intrinsic network may express the different way of information processing performed from the ventral compared with the dorsal segment of the hippocampus. It is hypothesized that functional specializations of basic local network parameters such as GABA_B_Rs and NMDARs support the distinct roles played by DH and VH networks. Adaptive modifications in other parameters of the neural circuitry contribute to keeping excitation/inhibition balance into the homeostatically regulated physiological range.
